# Exploring the microbial biotransformation of extraterrestrial material on nanometer scale

**DOI:** 10.1038/s41598-019-54482-7

**Published:** 2019-12-02

**Authors:** Tetyana Milojevic, Denise Kölbl, Ludovic Ferrière, Mihaela Albu, Adrienne Kish, Roberta L. Flemming, Christian Koeberl, Amir Blazevic, Ziga Zebec, Simon K.-M. R. Rittmann, Christa Schleper, Marc Pignitter, Veronika Somoza, Mario P. Schimak, Alexandra N. Rupert

**Affiliations:** 1 Extremophiles/Space Biochemistry Group, Department of Biophysical Chemistry, University of Vienna, Althanstrasse 14, A-1090 Vienna, Austria; 2 Natural History Museum, Burgring 7, A-1010 Vienna, Austria; 3 Graz Centre for Electron Microscopy, Graz, Austria; 4 Unité Molécules de Communication et Adaptation des Microorganismes (MCAM), Muséum National d’Histoire Naturelle, CNRS, 57 rue Cuvier, 75005, Paris, France; 5 Department of Earth Sciences, Western University, London, UK; 6 Archaea Biology and Ecogenomics Division, Department of Ecogenomics and Systems Biology, University of Vienna, Althanstraße 14, A-1090 Vienna, Austria; 7 Department of Physiological Chemistry, Faculty of Chemistry, University of Vienna, Althanstraße 14, 1090 Vienna, Austria; 8 Max Planck Institute for Marine Microbiology, Celsiusstrasse 1, D-28359 Bremen, Germany; 9 Department of Lithospheric Research, University of Vienna, Althanstrasse 14, A-1090 Vienna, Austria

**Keywords:** Microbiology, Astrobiology

## Abstract

Exploration of microbial-meteorite redox interactions highlights the possibility of bioprocessing of extraterrestrial metal resources and reveals specific microbial fingerprints left on extraterrestrial material. In the present study, we provide our observations on a microbial-meteorite nanoscale interface of the metal respiring thermoacidophile *Metallosphaera sedula*. *M. sedula* colonizes the stony meteorite Northwest Africa 1172 (NWA 1172; an H5 ordinary chondrite) and releases free soluble metals, with Ni ions as the most solubilized. We show the redox route of Ni ions, originating from the metallic Ni° of the meteorite grains and leading to released soluble Ni^2+^. Nanoscale resolution ultrastructural studies of meteorite grown *M. sedula* coupled to electron energy loss spectroscopy (EELS) points to the redox processing of Fe-bearing meteorite material. Our investigations validate the ability of *M. sedula* to perform the biotransformation of meteorite minerals, unravel microbial fingerprints left on meteorite material, and provide the next step towards an understanding of meteorite biogeochemistry. Our findings will serve in defining mineralogical and morphological criteria for the identification of metal-containing microfossils.

## Introduction

The ability of chemolithotrophic microorganisms to catalyze redox transformations of metals is an exquisite tool for energy transduction between a mineral body and a living entity. The different types of meteorites from diverse parental bodies (asteroids, Moon, or Mars) represent exceptional metal-bearing substrates that have experienced an exposure to multiple extreme conditions during their interstellar or interplanetary travel. During their journey in space, these meteoroids are constantly exposed to vacuum, radiation, and extreme temperature fluctuations. While microbial-mineral interfaces have been extensively investigated and microbial-mediated processing of metal-bearing terrestrial minerals has been abundantly harnessed, exploitation of meteorite resources requires detailed investigations of microbe-extraterrestrial mineral interactions. The study of meteorite-associated physiology of chemolithotrophic microbes is of a special focus due to implications for assessing the potential of extraterrestrial materials as a source of accessible nutrients and energy on the early Earth. Meteorites may have delivered a variety of essential compounds facilitating the evolution of life, as we know it on Earth^1–3^. Moreover, assessing the biogenicity based on extraterrestrial materials provides a valuable source of information for exploring the putative extraterrestrial bioinorganic chemistry that potentially might have occurred in the Solar System. Given that returned extraterrestrial mineralogical samples are to date inaccessible, meteorites are “space probes” available on Earth. The compositional variety of meteorites is very broad; over 400 minerals have been found in meteorites, predominantly troilite, olivine, pyroxene, feldspar, phyllosilicates, magnetite, kamacite, and taenite. The primitive (chondritic) classes of meteorites are the most abundant, with iron and nickel as the dominant ferrous metals^4^. Troilite [FeS] and FeNi alloys [Fe1-xNix] as meteoritic components can be potentially used as electron donors by iron and sulfur oxidizing organisms. Several laboratory experiments on meteorites have been performed, demonstrating that some iron-oxidizing bacteria (*e.g.*, *Leptospirillum ferrooxidans*, *Acidithiobacillus ferrooxidans*) could propagate on metal-bearing meteorite materials and use them as energy sources^5,6^. Recently, an *in situ* assessment of the microbial communities, which colonize stony meteorites, has been published, aiming to reveal metal-cycling microorganisms naturally contaminating astromaterials rich in FeNi-alloys and FeS^7^. The extreme thermoacidophile *Metallosphaera sedula* is a metallophilic archaeon that lives in hot acidic conditions (73 °C and pH 2) and uses various metal-containing ores to run its respiratory electron transport chain^8–10^. Previously, we have shown that this metal mobilizing archaeon can grow on Martian regolith simulants, actively colonizing these artificial extraterrestrial materials, releasing free soluble metals into the leachate solution and leaving specific spectral fingerprints^11^. In the present work, we explore the physiology and metal-microbial interface of *M. sedula*, living on and interacting with a real extraterrestrial material, H5 ordinary chondrite Northwest Africa 1172 ((NWA 1172), Supplementary Fig. 1)^12^. Specific chemical analysis of the meteorite-microbial interface at nm-scale spatial resolution allowed us to trace the trafficking of meteorite inorganic constituents into a microbial cell and to investigate iron redox behavior. Combining several analytical spectroscopy techniques with transmission electron microscopy (TEM) analysis, we provide a set of biogeochemical fingerprints left upon *M. sedula* growth on the NWA 1172 meteorite.

## Results and Discussion

### Chemolithotrophic growth of *M. sedula* on NWA 1172

**Figure 1:**
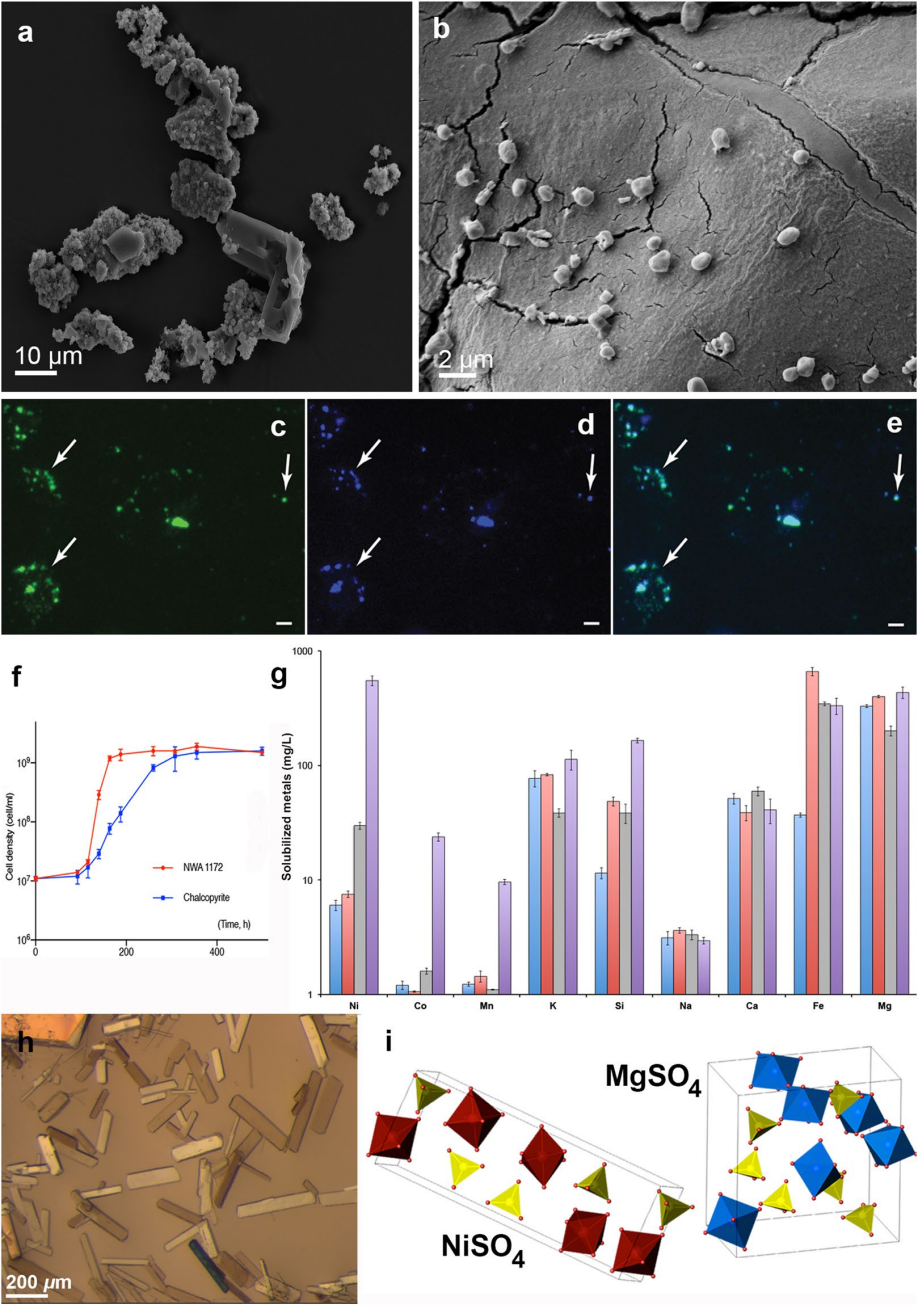
Biotransformation of the chondrite meteorite NWA 1172 by *M. sedula*. (**a**) Scanning electron microscopy (SEM) image of fragments of the chondrite meteorite NWA 1172 bioprocessed by *M. sedula*. (**b**) SEM image showing *M. sedula* cells colonizing the surface of the meteorite particles. **(c–e**) Multi-Labeled-Fluorescence *in situ* Hybridization (MiL-FISH) of *M. sedula* cells grown on NWA 1172 as the sole energy sources: (**c**) MiL-FISH images of cells (green) after hybridization with the specific oligonucleotide probe targeting *M. sedula*; (**d**) DAPI staining of the same field (blue); (**e**) Overlaid epifluorescence image, showing overlap of the specific oligonucleotide probe targeting *M. sedula* with DAPI signals. Arrows indicate cells of *M. sedula*. Scale bar, 2 µm. (**f**) Growth curves of autotrophic cultures of *M. sedula* cultivated at 73 °C on NWA 1172 (red) and chalcopyrite (blue). Legends represent the corresponding type of energy source. (**g**) Inductively coupled plasma-optical emission spectrometry (ICP-OES) analysis of released metal ions in the supernatant of *M. sedula* cultures grown on NWA 1172 as the sole energy source. Samples were taken at “0” time point (red), from late exponentially growing cultures of *M. sedula* (purple), and from corresponding abiotic controls (blue and grey, respectively). (**h**) Single crystals of nickel sulfate hexahydrate and magnesium sulfate heptahydrate were obtained after recrystallization of the crystalline material (α) shown in Supplementary Fig. 4. (**i**) Atomic structures of NiSO_4_ × 6 H_2_O and MgSO_4_ × 7 H_2_O from crystals in (**h**) as investigated with single crystal X-ray diffraction, with the unit cell for each structure represented. Crystal water has been removed for clarity. Legend: Ni(H_2_O)_6_, red octahedra; Mg(H_2_O)_6_, blue octahedra; SO_4_^2−^, yellow tetrahedra. Points and error bars show the mean and error-represented standard deviation, respectively, of n = 3 biological replicates. If not visible, error bars are smaller than symbols.

*M. sedula* was capable of autotrophic growth on stony meteorite NWA 1172, utilizing metals trapped within it as the sole energy source. Scanning electron microscopy (SEM) investigations (Fig. 1a,b and Supplementary Fig. 2) and multi-labeled fluorescence *in situ* hybridization (MiL-FISH) with an *M. sedula*-specific ribosomal RNA-targeted probe (Fig. 1c–e, Supplementary Fig. 3, and Supplementary Table 1) indicate an active colonization of the meteorite material by *M. sedula*. As reported earlier, the round to slightly irregular coccoid cells of *M. sedula* possess pilus-like structures and can be motile when grown on the metal ore^9^. When grown in the presence of NWA 1172, cells of *M. sedula* were characterized by intensive vivid motility (Supplementary Movie 1). In comparison to chalcopyrite, *M. sedula* had a superior growth rate on the stony meteorite (Fig. 1f). Grown in the presence of chalcopyrite, cells of *M. sedula* displayed motility as well (Supplementary Movie 2). Meteorite-grown *M. sedula* reached stationary phase at 163 h (maximum density 18.5 ± 2 × 10^8^ cells/mL, a mean generation time of 8 h), while chalcopyrite-grown cells were still in exponential phase at that time point, and reached the stationary phase after 300 h (maximum density 15.1 ± 5 × 10^8^ cells/mL, a mean generation time of 21.4 h) (Fig. 1f). This difference reflects the beneficial contribution of NWA 1172 as the sole electron donor suggesting its preferential nature as energy source for *M. sedula*. NWA 1172 is a chondrite meteorite with relatively high iron abundance^12^ and a wide range of other metal elements (Supplementary Fig. 1). Those might be used by *M. sedula* as an energy source to satisfy its bioenergetic needs and as specific metabolic/enzymatic cofactors, providing more optimal constitutive and/or structural elements for enzymatic machinery. In addition, copper sulfides, such as chalcopyrite, dictate certain inevitable issues in respect with metal mobilizing due to their refractory characteristics. Hence, the porosity of NWA 1172 (typically of a few percent^13^) might also reflect the superior growth rate of *M. sedula* over refractory and densely packed chalcopyrite.

As a result of tight biogeochemical interactions, by means of its metal oxidizing machinery^14–16^, *M. sedula* released free soluble metals (Ni, Si, Co, Mn, and K) into the cultivation medium (Fig. 1g). Ni and Si were predominantly released, reaching 0.55 and 0.17 g/L, respectively, with lesser contributions from Co, Mn, and K ions (Fig. 1g). Dehydration-crystallization and X-ray diffraction experiments (Supplementary Fig. 4) revealed the chemical speciation of the released Ni: nickel sulfate hexahydrate^17^ in the tetragonal space group *P* 4_1_2_1_2 (a = 6.8 Å, b = 6.8 Å, c = 18.29999, α = β = γ = 90°) and magnesium sulfate heptahydrate^18^ in the orthorhombic space group *P* 2_1_2_1_2_1_ (a = 11.868 Å, b = 11.996 Å, c = 6.857 Å, α = β = γ = 90°), suggesting +2 oxidation state of extracted Ni ions and the occurrence of nickel sulfate in the leachate solution (Fig. 1i).

### Nanoanalytical spectroscopy of meteorite-microbial interface

**Figure 2:**
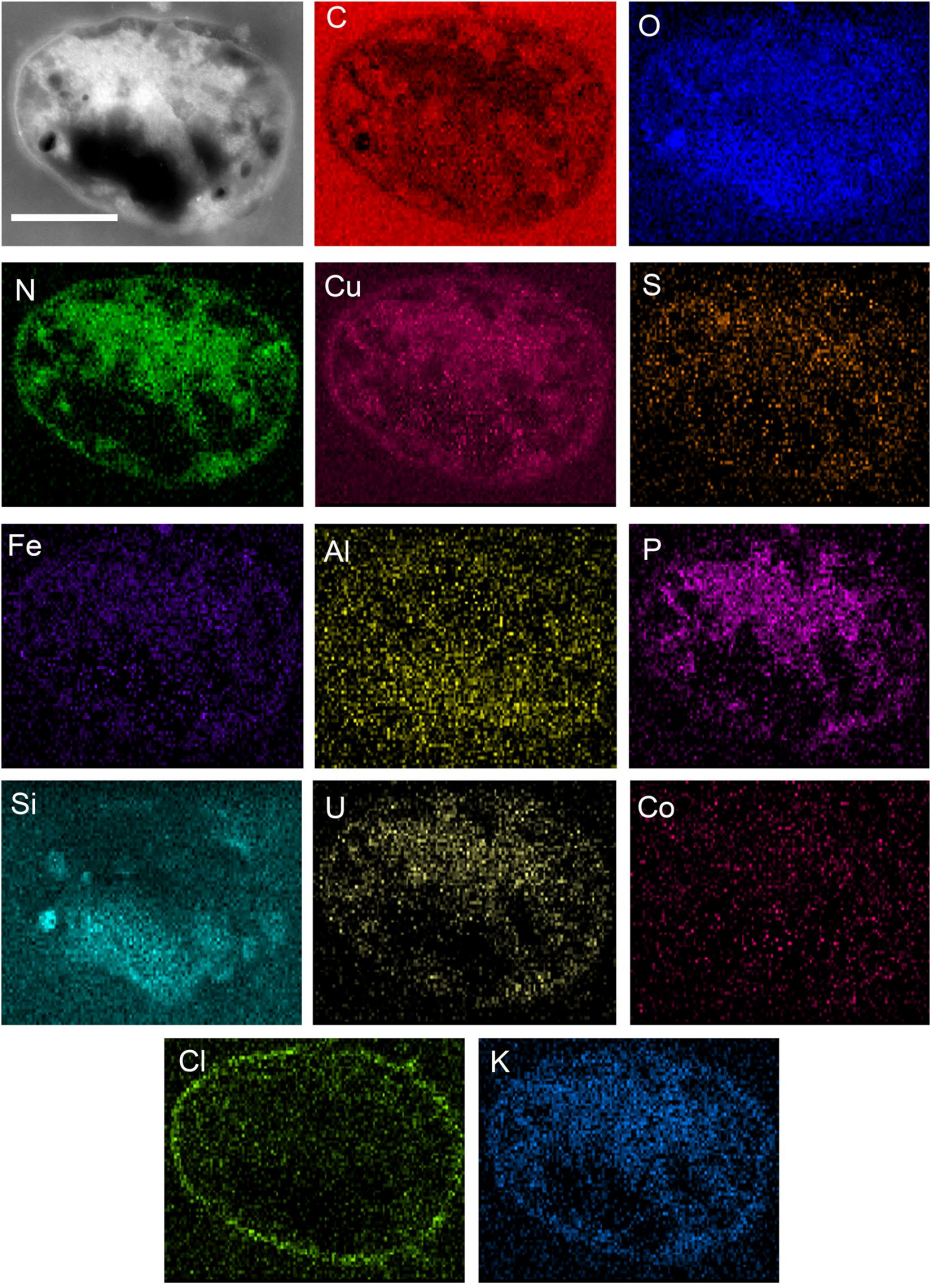
Elemental ultrastructural analysis of *M. sedula* grown on NWA 1172. The annular dark field (ADF) scanning transmission electron microscopy (STEM) image of a cell of *M. sedula* used for the EDS spectrum image acquisition and corresponding extracted carbon (C), oxygen (O), nitrogen (N), copper (Cu), sulfur (S), potassium (K), chlorine (Cl), iron (Fe), aluminum (Al), phosphorus (P), cobalt (Co), silicon (Si), and uranium (U) elemental maps. Scale bar, 0.5 µm.

Observations of ultra-thin sections of *M. sedula* cells grown on NWA 1172 revealed round-shaped, irregular cocci with a diameter around 1 µm (Fig. 2). These irregular coccoid morphologies were characterized by the presence of electron-dense dark areas along the cell envelope and extensive dark accumulations in the cytosol (Fig. 2). Elemental ultrastructural analysis of *M. sedula* grown on NWA 1172 was performed to investigate metal acquisition patterns of this archaeon, *i.e*., enabling us to verify the content and localization of metals in *M. sedula* (Fig. 2). The following observations were made using energy-dispersive X-ray spectroscopy (EDS) analysis performed in scanning transmission electron microscopy (STEM) mode: (1) the elemental maps show abundant C, O, N, S, Cu, P, Fe, Al, Co, and K content of *M. sedula* cells; (2) Cu, K, Cl, Fe, Al, and P signals were localized both on the cell surface and intracellularly; (3) C, O, and N were evenly distributed giving strong intracellular signals which likely arose from organic content (*e.g*., proteins and carbohydrates) present in *M. sedula* cells; (4) Si accumulations produced strong intracellular signals, which correspond to the dark electron dense areas of the TEM image (intracellular deposits); and (5) Co and K were evenly represented inside the cell; (Fig. 2, Supplementary Fig. 5).

The pronounced membrane-bound signal of Cu reflects the great diversity and active content of multi-copper oxidases widely distributed in *M. sedula* branched respiratory chains within a diverse number of terminal oxidases^10,14–16^. Apart from these multi-copper oxidases involved in iron and sulfur oxidative respiration network, membrane-associated specific Cu-transporters might also implement in Cu binding observed in Fig. 2 (Cu map). An efficient Cu transportation and sequestration system was described in *M. sedula*^19,20^. Incorporation of Fe and S may naturally speak on the account that these elements are potentially used as energy sources in oxidation process coupled to electron breathing (Fig. 2, Fe and S maps). Intracellular Si accumulations in form of bulk deposits comprise 5.9% of the overall elemental content, and suggest the beginning of silicification of *M. sedula* during growth on meteorite material (Fig. 2, Si map and Supplementary Table 2). Silicification processes have been shown for various microbial systems, including *in situ* studies in modern silicifying hydrothermal systems^21^ and laboratory investigations of artificial microbial silicification^22–24^, highlighting the potential of silica matrices for the morphological preservation of putative microfossils.

TEM analysis revealed the presence of extracellular vesicle-like morphologies (encapsulated round-shaped particles with an average diameter of 200 nm; Supplementary Fig. 6a). The monolayers of *M. sedula* vesicles have been shown to catalyze iron oxidation and solubilization of mineralized copper from chalcopyrite under the energy-limited lithoautotrophic conditions^25^. Apart from metal transformation, synthesis and secretion of membrane vesicles might serve as a general mechanism of extracellular metal sequestration by binding with chelating agents, *e.g*., proteins, and enzymatic detoxification of the metal to a less toxic form. The observed vesicles, formed during the growth of *M. sedula*, may be capable of mineral oxidation, contributing to metal biotransformation and potentially metal immobilization from NWA 1172. Our EDS analysis performed in STEM mode showed that these carbonaceous and oxygenated vesicles harbor Si, Ni, Cu, Fe, Cl, and Al (Supplementary Fig. 6a), which reflects their ability to immobilize metals from the meteorite material.

**Figure 3:**
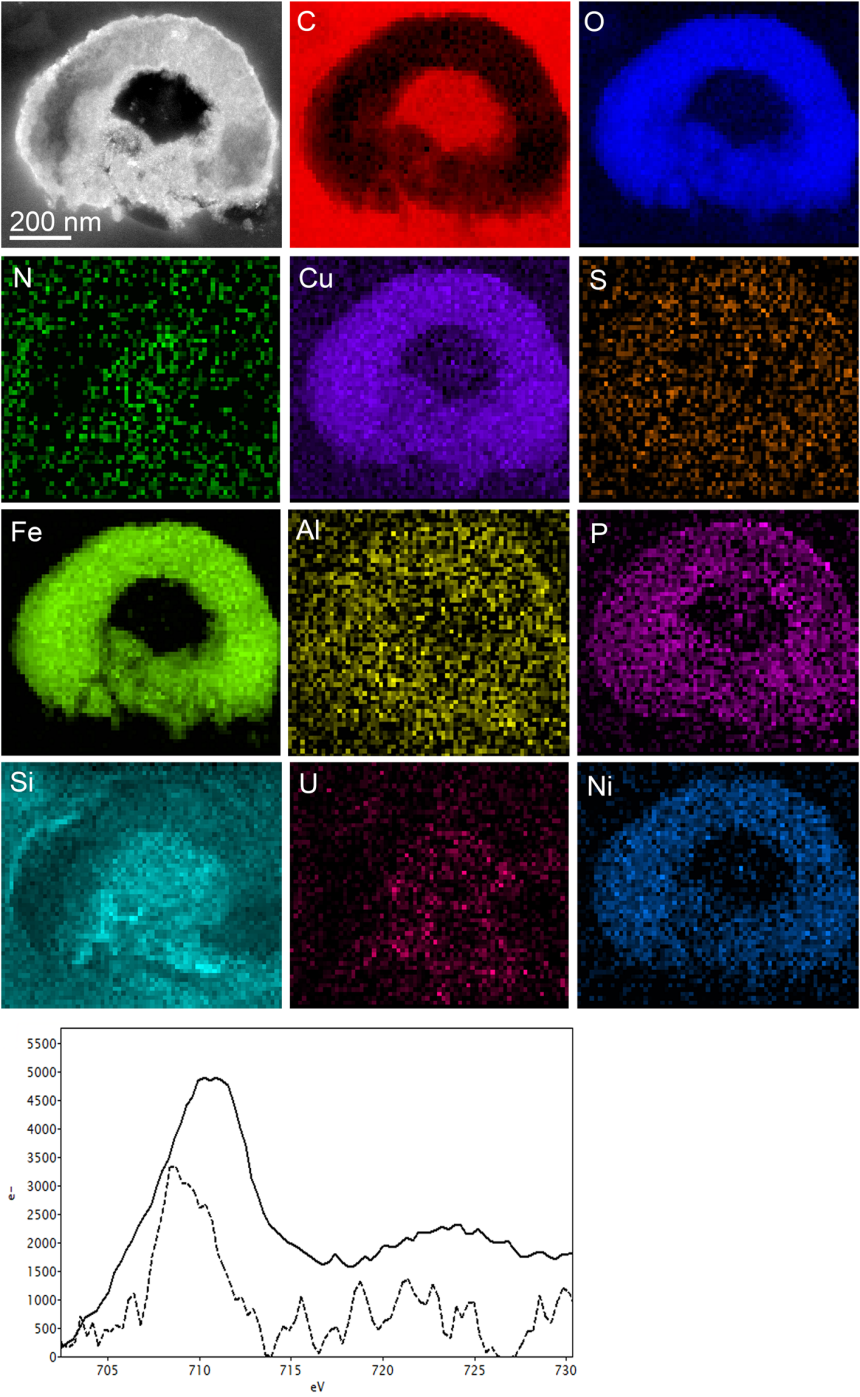
Elemental ultrastructural analysis of *M. sedula* empty envelopes encrusted during growth on NWA 1172 and corresponding Fe L_2,3_-edge core electron energy loss (EEL) spectra. The high angular annular dark field (HAADF) scanning transmission electron microscopy (STEM) image of a heavily encrusted cell remnants of *M. sedula* used for the EDS spectrum image acquisition and corresponding carbon (C), copper (Cu), phosphorus (P), iron (Fe) oxygen (O), nickel (Ni), sulfur (S), and nitrogen (N) elemental maps. Corresponding Fe L_2,3_-edge core electron energy loss (EEL) spectra acquired from the S-layer of *M. sedula* cells depicted in Supplementary Fig. 8a (shown as dotted line) and from the crust in Supplementary Fig. 8c (shown as solid line) are provided at the bottom panel.

Notably, TEM observations of cross-sections of *M. sedula* cells revealed the coexistence of cells in different stages of biomineralization: cells with not fully mineralized cell envelope (Fig. 2) and at more advanced steps of mineral formation as heavily encrusted mineralized cell remnants (Fig. 3 and Supplementary Figs. 7, 8). Elemental ultrastructural analysis of heavily encrusted cell remnants showed that the crust layer of variable thickness (up to 250 nm) was composed of Fe, Cu, Si, Al, Ni, S, C, N, O, and P (Supplementary Table 3). Further comparative electron energy loss spectra (EELS) analysis uncovered the spatial distribution and fine structure of iron species in heavily encrusted cell remains and on the cell surface of *M. sedula* (Fig. 3, bottom panel). The EELS measurements, acquired locally (point analysis with a beam diameter of 1 Å) in STEM mode, demonstrate that the cell surface of *M. sedula* is bearing a mixed valence iron distribution with dominant Fe^2+^ species (dotted line at Fig. 3, bottom panel). Fe^2+^ detected on the cell surface (Fe L_3_-edge at ~708 eV, Fig. 3, bottom panel) may primarily originate due to the leaching of Fe^2+^ from the meteorite. This spectrum also shows the minor presence of Fe^3+^ species (a shoulder of Fe L_3_-edge at ~710 eV) which can be explained by extracellular Fe^2+^ oxidation performed by a membrane bound iron oxidizing machinery of *M. sedula*, which is encoded by genes of the *fox* cluster^10,14–16^. A microbially replenished Fe^3+^ supply (in addition to abiotic Fe^3+^) ensures further meteorite oxidation as well as access to metals for the cells. The Fe^3+^ species detected on the cell surface of *M. sedula* can effectively function as an oxidizing agent for meteorite at the cell-meteorite interface. In addition to the direct *M. sedula* mediated biooxidation of metals, the involvement of abiotic factors may facilitate the process of metals mobilization from NWA 1172, representing an indirect mechanism of metal solubilization. Abiotic oxidizing attack of Fe^3+^ on the solid mineral enables the mobilization of metals from the solid matrix^26^, suggesting that both direct and indirect mechanisms contribute to the dissolution of metals mediated by *M. sedula*. The Fe L_2,3-_edges from heavily encrusted cell remnants show the predominant presence of Fe^3+^ species (solid line at Fig. 3, bottom panel, Fe L_3_-edge at ~710 eV), which can be explained by accomplished Fe^2+^ oxidation followed by cell encrustation and entombment in the mineralized form of a mixture of different amorphous iron oxides/hydroxides with the predominant form of Fe^3+^. Similar cell surface encrustation, but with tungsten crystalline nanolayers, we have previously shown for *M. sedula* grown on tungsten-bearing terrestrial materials^27,28^. In the case of our study with NWA 1172, TEM observations show that the encrusted cell remnants and iron bearing accumulations on the cell surface of *M. sedula* have an amorphous structure. Consequently, further studies were directed at spectral and mineralogical analysis of the meteorite surface after the exposure to *M. sedula*.

### Mineralogical and geochemical analyses of microbial fingerprints left on NWA 1172

**Figure 4:**
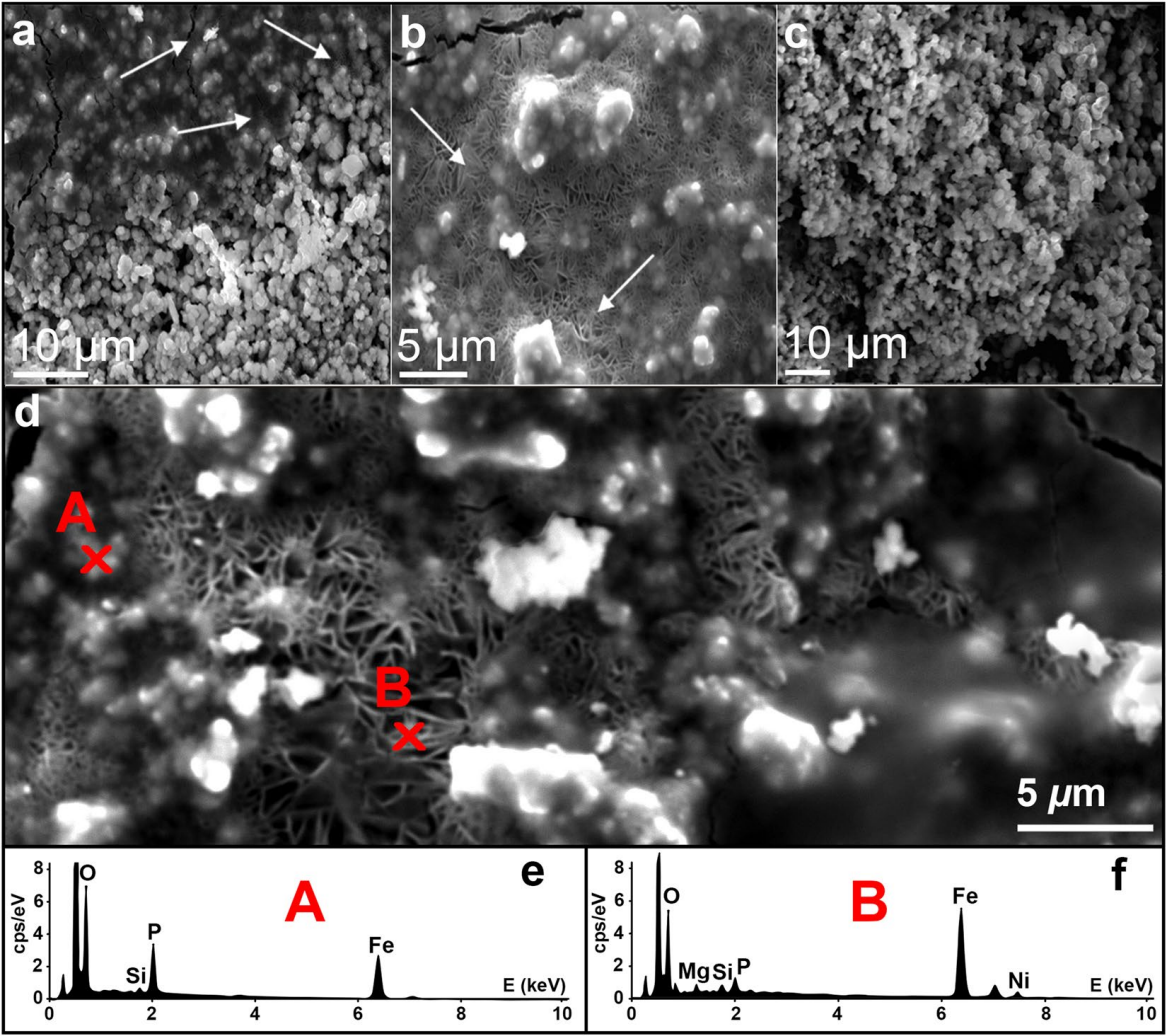
Alteration of the surface of the chondrite meteorite NWA 1172 slabs mediated by *M. sedula*. (**a**) Secondary electron (SE) image of a NWA 1172 slab cultivated with *M. sedula* at 73 °C. (**b**) Magnified SE image of a NWA 1172 slab cultivated with *M. sedula* at 73 °C. (**c**) SE image of abiotically exposed slab of NWA 1172 to the cultivation medium at 73 °C. (**d**) Magnified area of SE image of NWA 1172 slab cultivated with *M. sedula* at 73 °C. (**e**) EDS spectra of globular structures (marked with red A) that form iron oxides aggregates, containing mainly Fe and P. (**f**) EDS spectra of branched network of crystalline iron oxides (marked with red b). Arrows indicate the areas where crystalline iron oxides occur.

Investigations of the biologically mediated alteration of the meteorite surface by cultivation of *M. sedula* with slab fragments of NWA 1172 indicated the presence of globular iron-rich aggregates (with a size ranging from <0.5 to 2 μm for single globules) along with areas of crystalline iron oxides as a specific microbial alteration left upon *M. sedula* growth (Fig. 4a,b,d). Similar types of globular iron-rich aggregates were observed on the surface of the meteorite abiotically exposed to *M. sedula* medium at 73 °C, illustrating that they are the result of abiotic, *i.e*., chemically induced rusting processes (Fig. 4c). EDS analysis of these globular aggregates in chemically changed and biologically altered meteorite surfaces showed that they are dominated by Fe and P with a variable amount of S and Si (Fig. 4e). However, the iron oxides (as determined using EDS), which appear as branched porous network represented on Fig. 4b,d,f, occurred only along the meteorite surface exposed to *M. sedula*, suggesting that it is solely of biogenic origin. In a few previously published studies, iron meteorites and carbonaceous chondrite meteorites provided metal components as suitable energy substrates to maintain the bacterial growth and chemolithotrophic metabolism^5,6^. Similar near-spherical Fe-, P-, and S-containing aggregates along with needle-like crystals of iron oxides were observed during iron meteorite weathering by the iron-oxidizing acidophilic bacteria *A. ferrooxidans*^5^.

Micro X-ray diffraction (µXRD) data were acquired on a bioprocessed NWA 1172 slab fragment after the cultivation with *M. sedula*, and the following secondary oxides/alteration minerals were characterized: goethite, lepidocrocite, ferrihydrite, hematite, and maghemite (*i.e*., similar work conducted on an unprocessed meteorite slab shows that none of these minerals were originally present in the meteorite) (Supplementary Fig. 9). The most-abundant primary minerals in ordinary chondrites are olivine and enstatite (+/− plagioclase). These primary minerals are visible on every pattern in µXRD analysis performed in this study, as X-rays penetrate through the surface layer with microbial alterations. Occasionally, primary kamacite, troilite, and magnetite were also detected. Our µXRD measurements are in agreement with previously reported biogenic crystalline ferric iron (oxy)hydroxides, such as goethite and lepidocrocite for the iron oxidizing acidophile *Acidithiobacillus ferrooxidans* during iron meteorite weathering^5^ and closely related species *Sulfolobus acidocaldarius*, the only other member of the *Sulfolobales* for which such measurements are available^29^.

**Figure 5:**
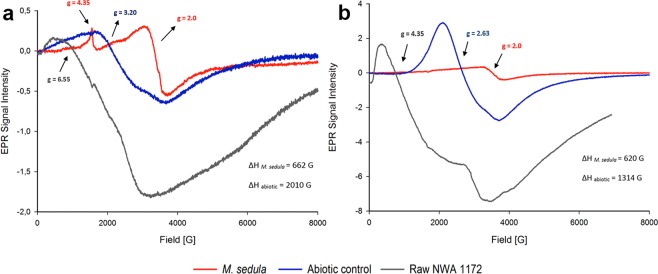
Electron Paramagnetic Resonance (EPR) spectra of raw NWA 1172 (gray line), NWA 1172 bioprocessed by *M. sedula* (red line) and NWA 1172 after the treatment with cultivation medium, but without *M. sedula* (abiotic control, blue line). (**a**) Spectra recorded at 90 K with assigned linewidth (deltaH) and g-values. (**b**) Spectra recorded at 273 K.

Electron Paramagnetic Resonance (EPR) measurements were performed to (1) identify paramagnetic species in NWA 1172 and to (2) investigate the impact of *M. sedula* on NWA 1172 with a possible effect on the oxidation state of paramagnetic species. Measurements were performed at 90 K (Fig. 5a) and 273 K (Fig. 5b) to increase the chance for unambiguous iron detection. Iron oxides are the predominant component (51%) of the chondrite NWA 1172 (Supplementary Fig. 1) and iron mixed paramagnetic Fe^3+^ species in high and low spin states in biogenic samples were identified via EPR. Accumulation of Fe^3+^ in the biogenic sample at 90 K (Fig. 5a, red line) was observed and might be the consequence of extensive Fe^2+^ oxidation of the minerals mediated by *M. sedula*. After cultivation with *M. sedula*, the biogenic sample shows a prominent sharp high spin Fe^3+^ signal with a g-value of 4.35 and a low spin Fe^3+^ signal at g-value 2 (Fig. 5a). Compared to the raw meteorite material, the appearance of a prominent high spin Fe^3+^ signal (a sharp peak with a g-value of 4.35) along with a low spin Fe^3+^ signal at g-value 2 in the biogenic sample may indicate an increase in Fe^3+^ species due to biooxidative activity of *M. sedula*. These characteristic sharp high spin and low spin Fe^3+^ signals are not represented in abiotically treated NWA 1172 (Fig. 5). The abiotic spectrum is characterized by a signal with a shifted g-value 3.20 and broad linewidth (ΔH = 2010 G), which might refer to multiple ionic mixtures. Interestingly, the spectral features of abiotically treated NWA 1172 closely resemble those of abiotically treated Martian regolith simulant S-MRS^11^. This abiotic peak at g-value 3.20 is not detectable anymore in biogenic samples of NWA 1172 (Fig. 5a, red curve) and S-MRS^11^. While the distinct high spin Fe^3+^ signal is not detectable in the biogenic sample when measured at 273 K (Fig. 5b), the low spin Fe^3+^ signal at g-value 2 appears after the cultivation showing us the presence of mineral transforming iron-oxidizing microorganisms.

## Conclusions

Our investigations provide the further step towards extending our knowledge of meteorite biogeochemistry. Heterogeneous iron species Fe^2+^/Fe^3+^ with dominant Fe^2+^ are represented on the cell surface, indicating active Fe redox processing at the cell surface of *M. sedula* and suggesting Fe oxidative solubilization of meteorite material. *M. sedula* releases free solubilized metals (Fig. 1g) and is capable of intracellular and cell surface incorporation of metals when grown on meteorite material (Figs. 2, 3, and 4). Meteorite biotransformation mediated by *M. sedula* leads to nickel solubilization (Fig. 1g–i) with the formation of nickel sulfate (NiSO_4_)-bearing material (Fig. 1h,i). Such nickel enrichment/NiSO_4_ deposition can be considered as a metabolic signature resulted from microbial leaching activity of meteorite minerals. The heavily metal encrusted cell remnants described in the present study and its fine structural details may constitute relevant biosignatures to be looked for in the geological record, if they are not destructed during diagenetic or metamorphic processes and are intact during different stages of fossilization. Our nanoanalytical spectroscopy investigations of heavily mineralized *M. sedula* cells grown on the NWA 1172 meteorite revealed the enrichment of Cu, Fe, O, and Si in the amorphous crust with a mixture of P, Ni, S and Al, which may likely correspond to the amorphous product Cu_x_Fe_y_O_z_(SPNiAl)–SiO_2_ formed on the late biomineralization stages of cells encrustation. Our work provides a deeper insight into the biologically-mediated processing of extraterrestrial material and implications for natural samples, representing a special interest for space exploration missions.

## Methods

### Cultivation of *M. sedula*

*M. sedula* (DSMZ 5348) cultures were grown aerobically in DSMZ88 *Sulfolobus* medium containing 1.3 g (NH4)_2_SO_4_, 0.28 g KH_2_PO_4_, 0.25 g MgSO_4_·7 H_2_O, 0.07 g CaCl_2_·2 H_2_O, and 0.02 g FeCl_3_·6 H_2_O dissolved in 1 L of water. After autoclaving, Allen’s trace elements solution was added to 1 L media resulting in 1.80 mg MnCl_2_·4 H_2_O, 4.50 mg Na_2_B_4_O_7_·10 H_2_O, 0.22 mg ZnSO_4_·7 H_2_O, 0.05 mg CuCl_2_·2 H_2_O, 0.03 mg Na_2_MoO_4_·2 H_2_O, 0.03 mg VSO_4_·2 H_2_O, and 0.01 mg CoSO_4_ final concentration. The pH was adjusted to 2.0 with 10 N H_2_SO_4_.

Cultivation of *M. sedula* was performed as described before^11^ in 1 L glassblower modified Schott-bottle bioreactors (Duran DWK Life Sciences GmbH, Wertheim/Main, Germany), equipped with a thermocouple connected to a heating and magnetic stirring plate (IKA RCT Standard/IKA C-MAG HS10, Lab Logistics Group GmbH, Meckenheim, Germany) for temperature and agitation control. The bioreactors were equipped with three 10 mL graduated glass pipettes, permitting carbon dioxide and air gassing (with the gas flow of 9 mL min^−1^, adjusted to five bubbles s^-1^ by using 8 mm valves (Serto, Frauenfeld, Switzerland)) and sampling of culture, respectively. The graduated pipettes used for gassing were connected by silicon tubing to sterile 0.2 µm filters (Millex-FG Vent filter unit, Millipore, Billerica, USA). The graduated pipettes used for sampling were equipped with a Luer-lock system in order to permit sampling with sterile syringes (Soft-Ject, Henke Sass Wolf, Tuttlingen, Germany). The offgas was forced to exit via a water-cooled condenser (Ochs GmbH, Bovenden, Germany). For the cultivations of *M. sedula* at 73 °C the temperature inside the bioreactors was controlled by electronic thermocouple via the heating and magnetic stirring plates. For chemolithoautotrophic growth cultures were supplemented with 10 g/liter either chalcopyrite (provided by E. Libowitzky from the mineral collection of the Department of Mineralogy and Crystallography, University of Vienna) or NWA 1172 meteorite (provided by the NHM, Vienna). The minerals were ground and temperature sterilized at 180 °C in a heating chamber for a minimum of 24 hours prior to autoclavation at 121 °C for 20 min. When intact, rectangular slabs of NWA 1172 were used for fermentation, the aforementioned two steps sterilization procedure was applied, too. Abiotic controls consisting of culture media supplemented with sterilized NWA 1172 without *M. sedula* cells were included in the experiments. Growth of cells was monitored by phase contrast/epifluorescence microscopy and metal release; doubling times (in h) during exponential phase growth were calculated from the slopes of the growth curves based on time required for 2-fold increase of cell number. For the visualization of cells wiggling on solid particles they were stained by a modified “DAPI” (4′–6′- Diamidino-2-phenylindole) procedure^30^, observed and recorded with ProgRes® MF cool camera (Jenoptik) mounted on Nikon eclipse 50i microscope, equipped with F36–500 Bandpass Filterset (ex, 377/50 nm; em, 447/60 nm).

### Multi-labelled-Fluorescence *in Situ* Hybridization (MiL-FISH)

*M. sedula* cells were fixed in 2% paraformaldehyde (PFA) at room temperature for 1 hour, washed three times in distilled water, centrifuged at 10,000 rpm and stored in 50:50, ethanol:PBS (phosphate buffer saline). A 16 S rRNA phylotype specific probe for *M. sedula* was designed with the software package ARB^31^ and labeled with 4x Atto488 via Click chemistry (biomers.net GmbH, Ulm, Germany) (Supplementary Table 1). Fixed cells were mounted on 10 well Diagnostica glass slides (Thermo Fisher Scientific Inc. Waltham, USA) and MiL-FISH conducted directly on them after Kölbl *et al*.^11^. Cells were hybridized with 30% formamide for 16 hours. For experimental positive controls *Gramella forsetii* strain KT0803 was hybridized with a 4x labelled general bacterial probe EUB338. Positive control for the specificity of the phylotype specific probe M.sedula_174 was given by including *M. sedula* DSM5348 in all experiments. After hybridization slides were washed for 15 minutes at 48 °C (14 to 900 mM NaCl, 20 mM Tris-HCl [pH 8], 5 mM EDTA [pH 8], and 0.01% SDS) at a stringency adjusted to the formamide concentration used. Cells were counterstained by incubation for 10 min with 10 mg ml^−1^ DAPI followed by rinsing in distilled water 3 times before CitiFluor (CitiFluor Ltd., London, England) mounting medium was applied to slides with a coverslip. Fluorescence images were taken with an AxioCam Mrm camera mounted on an Axioscope2 epifluorescence microscope (Carl Zeiss AG, Oberkochen, Germany) equipped with F36-525 Alexa 488 (ex, 472/30 nm; em, 520/35 nm) filter cube. Images were recorded with the PC-based AxioVision (release 4.6.3 SP1) imaging software.

### Metal analysis

To determine the extracellular concentrations of metal ions mobilized from the sulfide ores and from NWA 1172, culture samples were clarified by centrifugation. Samples of the resulting supernatants were filtered (0.44 µm pore size) and analyzed by inductively coupled plasma-optical emission spectrometer (ICP-OES) Perkin Elmer Optima 5300 DV. All reported values are averages from triplicate samples.

### Dehydration/Crystallization experiments

Cultures of *M. sedula* autotrophically grown on meteorite were harvested at early stationary phase, spread evenly on glass plates, and dehydrated within 60 days under oxic conditions at room temperature (Ø 7 cm, VWR International). Abiotic controls consisting of uninoculated culture media were included in all the experiments. Dehydration of cultures by slow evaporation led to the formation of a crystalline and an amorphous phase. The crystalline phase was isolated and recrystallized in aqueous solution. Single crystals were mounted on loops and investigated using a Bruker D8 Venture, equipped with multilayer monochromator, INCOATEC microfocus sealed tube (λ (MoKα) = 0.71073 Å) and CMOS Photon Detector.

### Preparation and characterization of NWA 1172 meteorite samples

A partially fusion crusted NWA 1172 meteorite sample with fresh broken surfaces was selected for the current study (Supplementary Fig. 1). This sample was selected because it represents a very pristine example of an H5 type ordinary chondrite. Even it is not an observed fall meteorite, it is classified as W0, thus corresponding to the least weathered (or more pristine) type of meteorite (*i.e.*, with no visible oxidation of metal or troilite; see, *e.g*., Wlotzka, 1993)^32^. The classification as W0 was further confirmed during our preliminary optical microscope observations (*i.e.*, thin sections of this meteorite).

After cutting off the fusion crusted parts of the sample, rectangular slabs with dimensions of about 40 × 23 × 10 mm and about 30 g each were prepared (the rest of the sample was used for the preparation of polished thin sections and for the experiments with crushed material). One of the two large surfaces of all the prepared slabs was then polished, the other five faces staying “raw” (*i.e.*, cut surfaces).

After completion of the experiments, the slabs were withdrawn from 1 L glassblower modified Schott-bottle bioreactors and characterized with SEM. SEM characterization was conducted at the Natural History Museum (Vienna, Austria) using a JEOL JSM 6610-LV with the operating conditions of 15 kV accelerating voltage and 0.1 nA beam current.

### Scanning electron microscopy

Cells of *M. sedula* harvested at stationary phase were prepared for electron microscopy by fixing in a solution of 1 vol.% glutaraldehyde in Na-Cacodylate buffer. Samples were dehydrated in a graded series of ethanol solutions and dried chemically using Hexamethyldisilazan (HMDS). Fixed samples were mounted on aluminum stubs, sputter-coated with Au, and examined at the University of Vienna with a Zeiss Supra 55 VP scanning electron microscope operated at 5 kV.

### Transmission electron microscopy

Cells of *M. sedula* harvested at stationary phase were primary fixed at 4 °C in a 1 M Na-Cacodylate buffer containing 2.5% glutaraldehyde. After primary fixation cells were post-fixed for 2 h in 1% OsO_4_. Cells were washed three times (2 × 0.1 M Na-cacodylate, 1x dH_2_O) and subsequently dehydrated by a gradual ethanol series (30%, 50%, 70%, 90%, abs.), each step with an incubation time of 30 minutes. Cells were spun down after each dehydration step for another 30 minutes and resuspended for the following ethanol treatment. Samples were embedded in Spurr Low Viscosity Resin (Electron Microscopy Sciences, United States) and polymerized at 60 °C for a minimum of 48 h. Semi- and ultrathin sectioning were performed via a Reichert-Jung Ultracut E ultramicrotome, 50–70 nm ultrathin sections were deposited on formvar/carbon-coated 200 mesh copper grids (Agar Scientific, UK).

High resolution STEM investigations were performed at the Graz Centre for Electron Microscopy (FELMI-ZFE, Graz), on a probe corrected FEI Titan G2 60–300 (S/TEM) microscope with an X-FEG Schottky field-emission electron source operated at 60 and 300 kV (current of 150 pA, beam diameter of 1 Å). The microscope is equipped with a FEI Super-X detector (Chemi-STEM technology), consisting of four separate silicon drift detectors and a Dual EELS - Gatan Imaging Filter (GIF) Quantum. High Angular Annular Dark Field (HAADF) and Annular Dark Field (ADF) detectors were used to acquire micrographs. Scanning transmission electron microscopy (STEM) and analytical spectroscopy by using electron energy loss (EELS) and dispersive X-ray (EDS) Spectrum Images were carried out for different areas of *M. sedula* cells. For each area, elemental maps were extracted from EELS and EDS spectrum images. Further, EELS and EDS spectra from representative areas on the cell surface and inside the cells have been acquired/extracted. The images and spectra were processed with Gatan’s Digital Micrograph being corrected for dark current and gain variations. Element quantification for EDS spectra was performed by using the k-factor method^33,34^.

### Micro X-ray diffraction analysis

Data were collected on a Bruker D8 Discover at the University of Western Ontario (London, Ontario, Canada), having θ-θ geometry, operating at 35 kV, and 45 mA with a radiation source of CoKα (1.79026 Å), and a Göbel mirror with a 300 μm pinhole collimator. A HI-STAR detector with General Area Detector Diffraction System (GADDS; Bruker-AXS 2010) software was used^35^.

### Electron Paramagnetic Resonance spectroscopy

The Electron Paramagnetic Resonance (EPR) spectra were recorded as described earlier (11) at the University of Vienna on an X-Band Bruker Elexsys-II E500 CW-EPR spectrometer (Bruker Biospin GmbH, Rheinstetten, Germany) at 90 ± 1 and 293 ± 1 K using a high sensitivity cavity (SHQE1119). Solid state EPR measurements were performed setting microwave frequency to 9 GHz, modulation frequency to 100 kHz, center field to 6000 G, sweep width to 12000 G, sweep time to 335.5 s, modulation amplitude to 20.37 G, microwave power to 15 mW, conversion time to 81.92 ms, and resolution to 4096 points. The samples were put in EPR quartz tubes (Wilmad-LabGlass, Vineland, NJ, United States) and scanned three times, of which the average was used for analysis. The spectrum of an empty control tube was subtracted from all sample spectra. All spectra were analyzed with the Bruker Xepr software.

## Supplementary information

Supplementary Information

Motility of <i>M. sedula</i> cells grown on the NWA 1172 meteorite as the sole energy source at 73°C after visualization by a modified DAPI fluorescence staining procedure.

Motility of <i>M. sedula</i> cells grown on chalcopyrite as the sole energy source at 73°C after visualization by a modified DAPI fluorescence staining procedure.
