# Supplementary material for: Exploring the microbial biotransformation of extraterrestrial material on nanometer scale
Source: Sci Rep. 2019 Dec 2;9:18028. doi: 10.1038/s41598-019-54482-7 (PMC6889503; doi:10.1038/s41598-019-54482-7)
Supplement: Supplementary file 1 — Supplementary Information [file 41598_2019_54482_MOESM1_ESM.pdf]

Exploring the microbial biotransformation of extraterrestrial material on nanometer scale

Tetyana Milojevic*, Denise Kölbl, Ludovic Ferrière, Mihaela Albu, Adrienne Kish, Roberta L. Flemming, Christian Köberl, Amir Blazevic, Ziga Zebec, Simon K.-M. R. Rittmann, Christa Schleper, Marc Pignitter, Veronika Somoza, Mario P. Schimak, and Alexandra N. Rupert

*Corresponding author: Tetyana Milojevic (tetyana.milojevic@univie.ac.at)

Supplementary Information:

Supplementary Figure 1: Macrophotographs and X-ray fluorescence analysis of the stony meteorite NWA 1172.

Supplementary Figure 2: Scanning electron microscopy images of the stony meteorite NWA 1172 bioprocessed by *M. sedula*.

Supplementary Figure 3: Phase contrast and fluorescence micrographs of cells of *M. sedula*.

Supplementary Figure 4: Crystalline material formed in cultures of *M. sedula* dehydrated by slow evaporation.

Supplementary Figure 5: Analytical spectroscopy of *M. sedula* cell grown on NWA 1172.

Supplementary Figure 6: The high angular annular dark field (HAADF) scanning transmission electron microscopy (STEM) images of extracellular vesicle-like morphologies in cultures of *M. sedula* grown on NWA 1172.

Supplementary Figure 6: Percentage elemental composition of *M. sedula* cell grown on NWA 1172.

Supplementary Figure 7: Elemental ultrastructural analysis of *M. sedula* empty envelope encrusted during growth on NWA 1172.

Supplementary Figure 8: Analytical nanospectroscopy of *M. sedula* grown on NWA 1172.

Supplementary Figure 9: µXRD patterns of the stony meteorite NWA 1172 slab altered by *M. sedula*.

Supplementary Table 1: Oligonucleotide probes used in this study.

Supplementary Table 2: Percentage elemental composition of *M. sedula* cell grown on NWA 1172 (calculated based on STEM-EDS analysis).

Supplementary Table 3: Percentage elemental composition of *M. sedula* empty envelope encrusted during growth on NWA 1172 (calculated based on STEM-EDS analysis).

Supplementary Video 1: Motility of *M. sedula* cells grown on the NWA 1172 meteorite as the sole energy source at 73°C after visualization by a modified DAPI fluorescence staining procedure.

Supplementary Video 2: Motility of *M. sedula* cells grown on chalcopyrite as the sole energy source at 73°C after visualization by a modified DAPI fluorescence staining procedure.

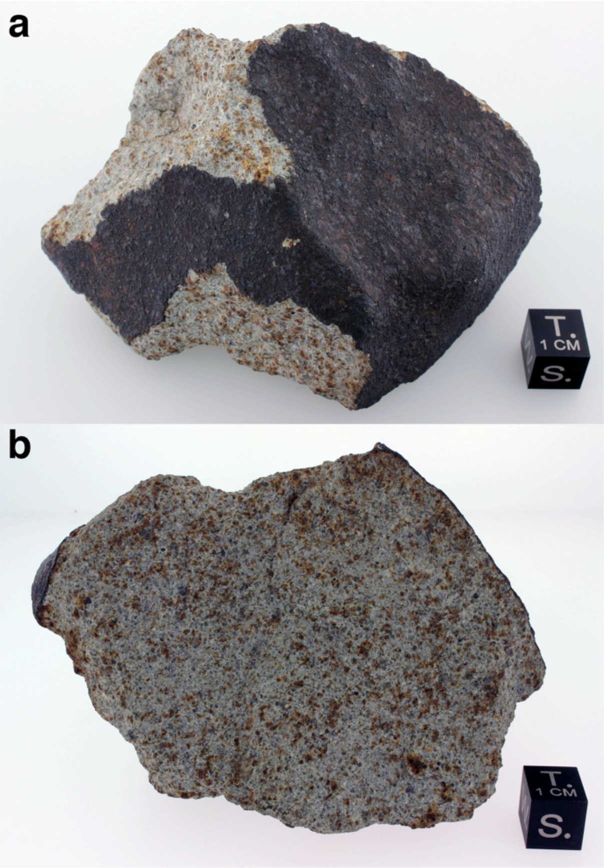

c

Element	Unit[1]	Results/data
Fe_2O_3	MA%	51.47
SiO_2	MA%	29.93
MgO	MA%	19.13
Na_2O	MA%	2.24
Al_2O_3	MA%	1.65
CaO	MA%	1.37
P_2O_5	MA%	0.20
SO_3	MA%	0.173
TiO_2	MA%	0.09
K_2O	MA%	0.08
Ni	µg/g	13491
Cr	µg/g	2896
Mn	µg/g	2053
Co	µg/g	1294
V	µg/g	58
Sc	µg/g	53
Cu	µg/g	46
Ba	µg/g	<27
Th	µg/g	15
As	µg/g	13
Zr	µg/g	10
Y	µg/g	9
Zn	µg/g	8
Rb	µg/g	7
Sr	µg/g	<6
Pb	µg/g	<4
Ga	µg/g	4
U	µg/g	3
Mo	µg/g	<2
Nb	µg/g	<2
LOI[2]	MA%	-8.41
total	MA%	99.92

[1]All concentration data in MA%.
[2]LOI = loss on ignition.
ISO 12677 = chemical analysis of refractory products by X-ray fluorescence (XRF) -- Fused cast-bead method.

Supplementary Figure 1. Macrophotographs and X-ray fluorescence analysis of the stony meteorite NWA 1172.

a, Macrophotograph of the chondrite meteorite NWA 1172 used in this study, showing a fresh dark black fusion crust and freshly broken surfaces. **b**, Inside of the NWA 1172 sample as seen on a cut surface during the preparation of the different slabs. **c,** Quantitative X-ray fluorescence (XRF) analysis from a fused bead of the NWA 1172 meteorite. Data are according to DIN/EN/ISO 12677 on 30 elements / element-oxides.

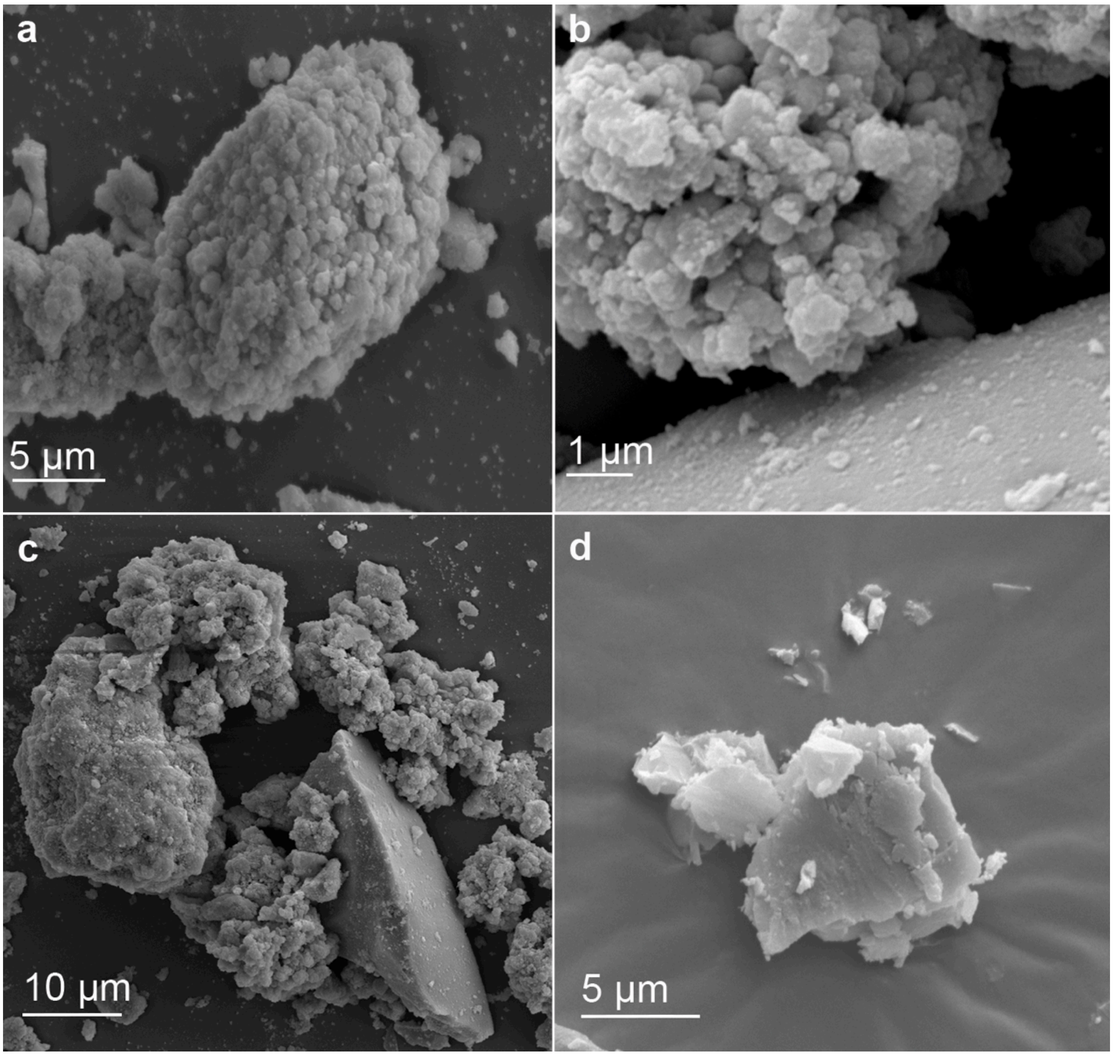

Supplementary Figure 2. Scanning electron microscopy images of NWA 1172 bioprocessed by *M. sedula*.

a-c, Scanning electron microscopy (SEM) images of fragments of the stony meteorite NWA 1172 after cultivation with *M. sedula*. **d**, SEM image of abiotically processed NWA 1172.

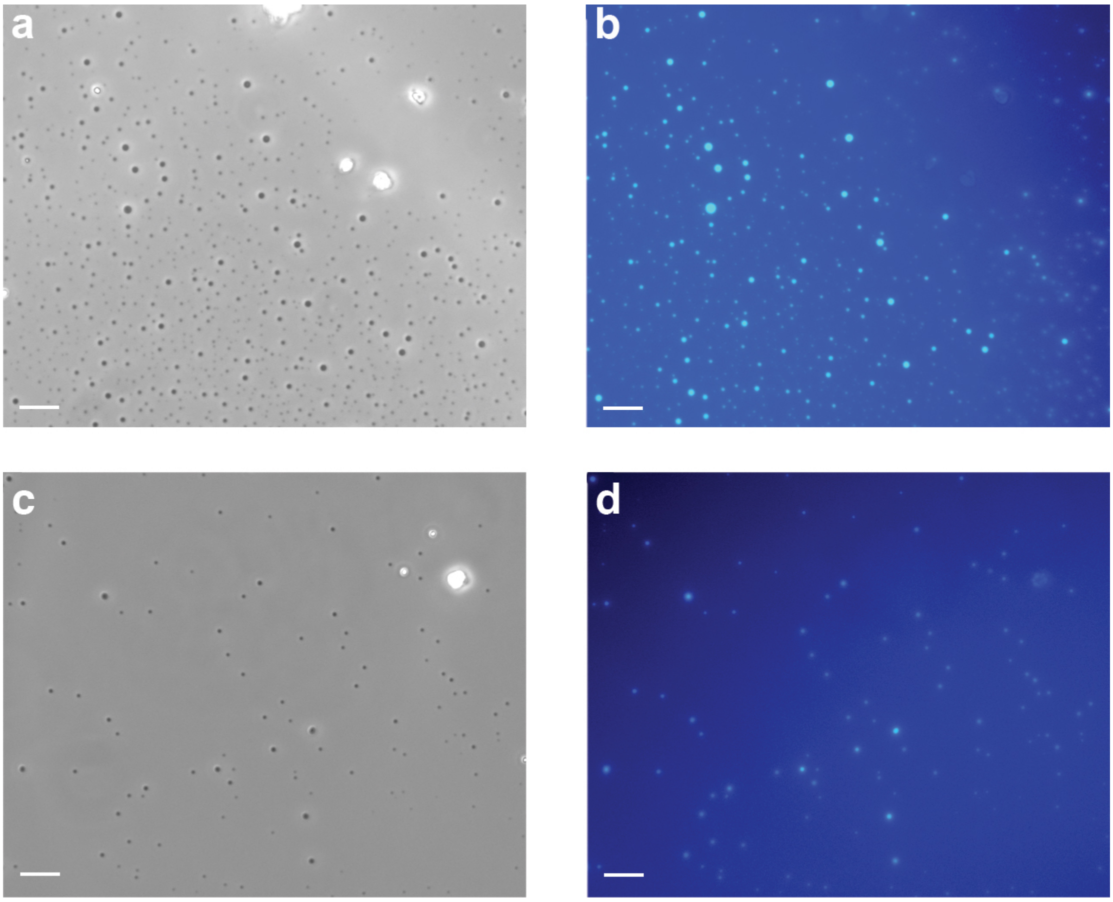

Supplementary Figure 3. Phase contrast and fluorescence micrographs of cells of *M. sedula*.

Phase contrast (**a, c**) and fluorescence micrographs (DAPI stained) (**b**, **d**) of cells of *M. sedula* grown on the NWA 1172 meteorite (**a, b**) and on chalcopyrite (**c, d**) at 73°C. Scale bar, 10 µm.

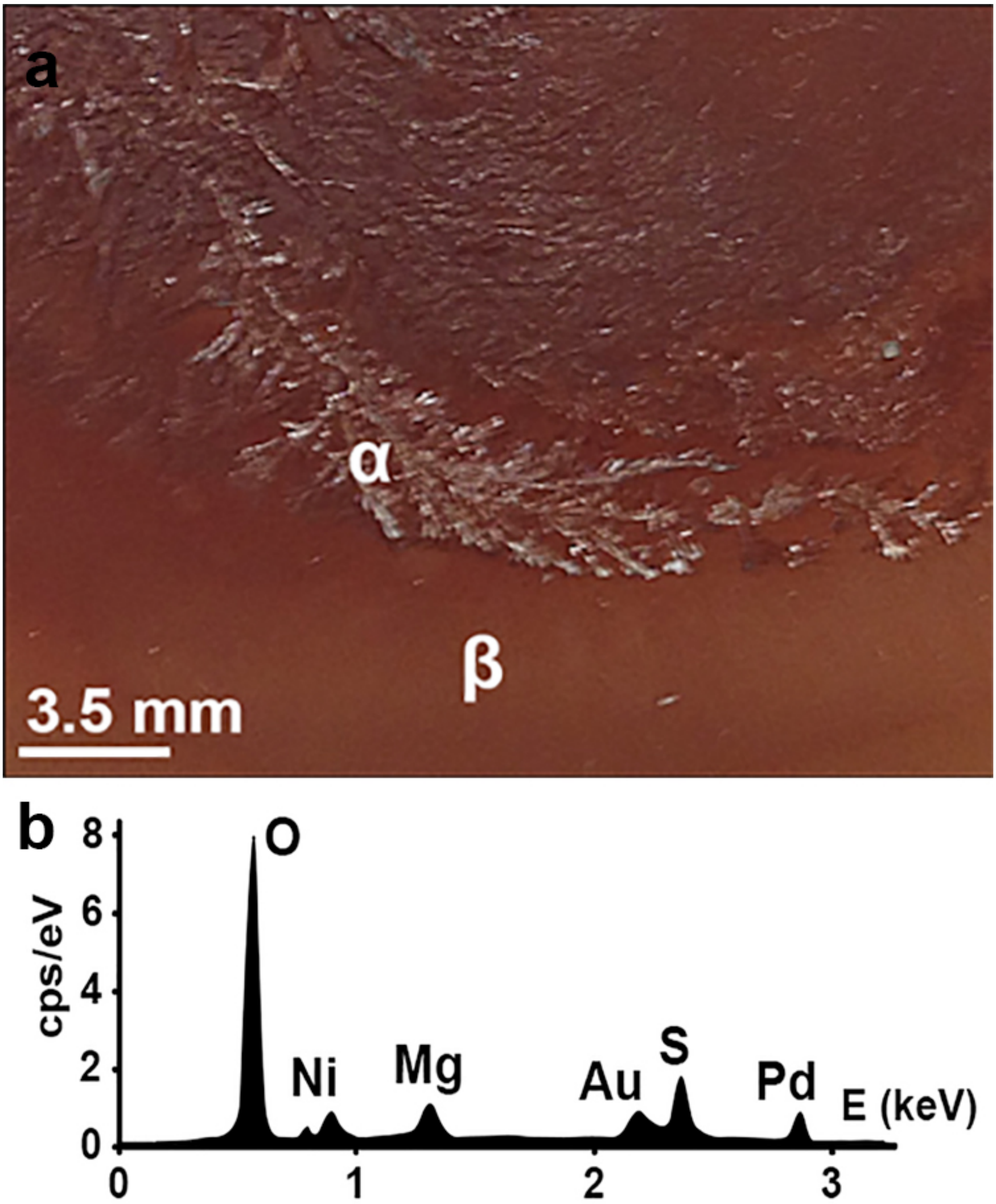

Supplementary Figure 4. Crystalline material formed in cultures of *M. sedula* dehydrated by slow evaporation. a, Macrophotograph of the crystalline material (α) and the amorphous precipitate (β) obtained after dehydration by slow evaporation of meteorite grown cultures of *M. sedula*. **b**, EDS analysis of the crystalline material (α) showing the content of Ni, O, S, and Mg ions.

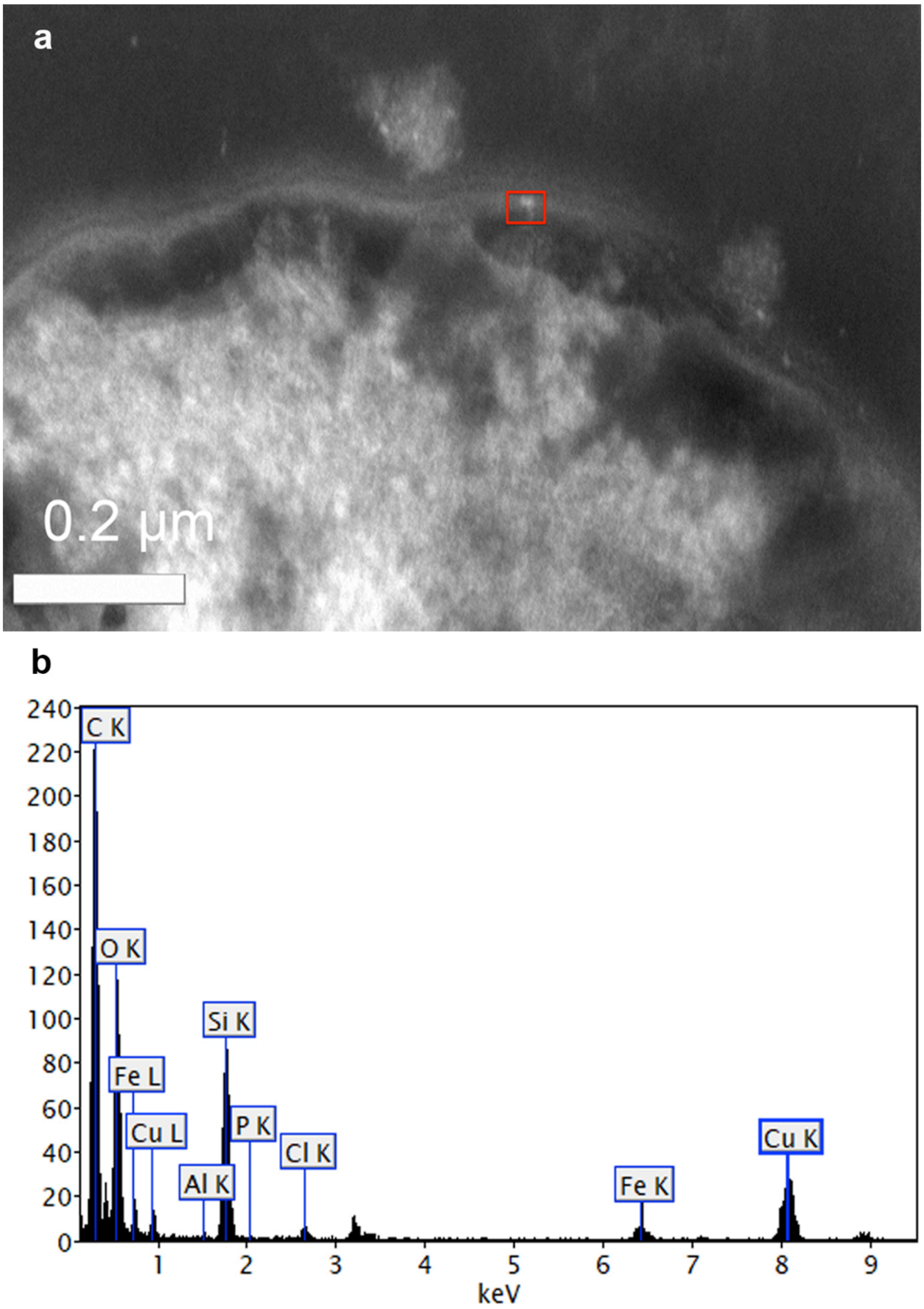

Supplementary Figure 5. Analytical spectroscopy of *M. sedula* cell grown on NWA 1172. a, The high angular annular dark field (HAADF) scanning transmission electron microscopy (STEM) image of the fragment of *M. sedula* cell used for analytical spectroscopy measurements. **b**, The energy-dispersive X-ray (EDS) spectra acquired from the area indicated on the HAADF-STEM image (**a**).

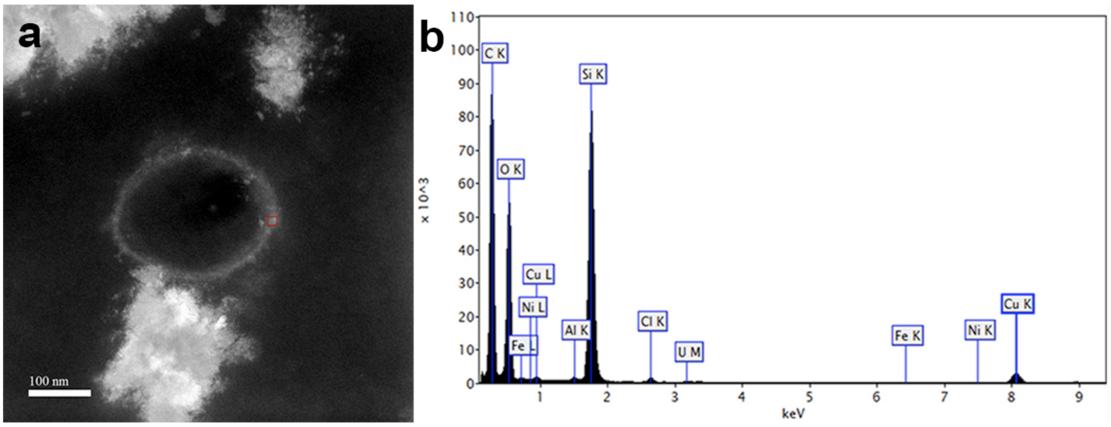

Supplementary Figure 6. The high angular annular dark field (HAADF) scanning transmission electron microscopy (STEM) images of extracellular vesicle-like morphologies in cultures of *M. sedula* grown on NWA 1172 (a) and corresponding STEM-EDS spectra (b) acquired from the point indicated on the HAADF-STEM image in (a).

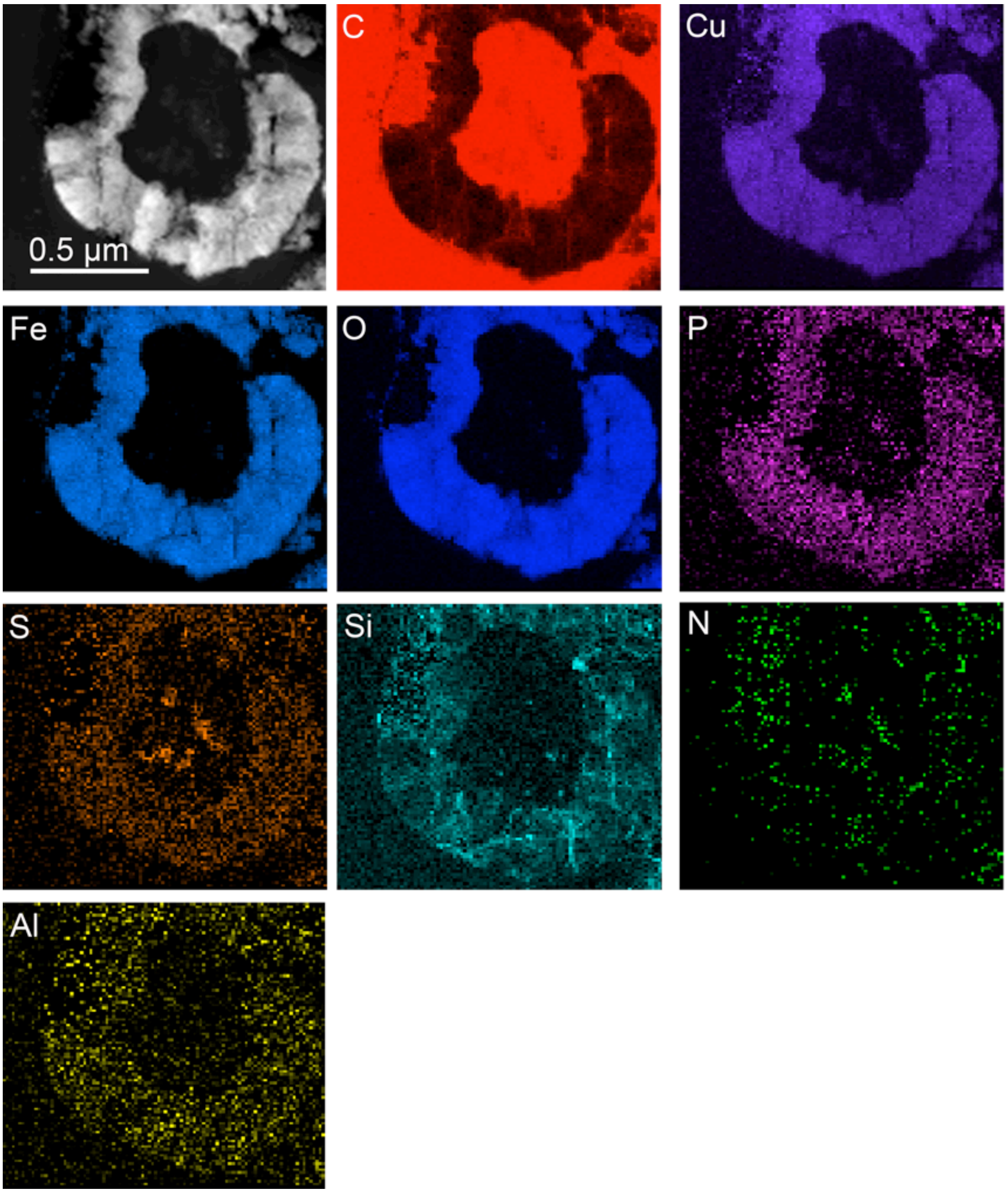

Supplementary Figure 7. Elemental ultrastructural analysis of *M. sedula* empty envelope encrusted during growth on NWA 1172.

The high angular annular dark field (HAADF) scanning transmission electron microscopy (STEM) image of a heavily encrusted cell remnants of *M. sedula* used for energy-filtered transmission electron microscopy (EFTEM) analysis and corresponding carbon (C), copper (Cu), phosphorus (P), iron (Fe) oxygen (O), nickel (Ni), sulfur (S), and nitrogen (N) elemental maps.

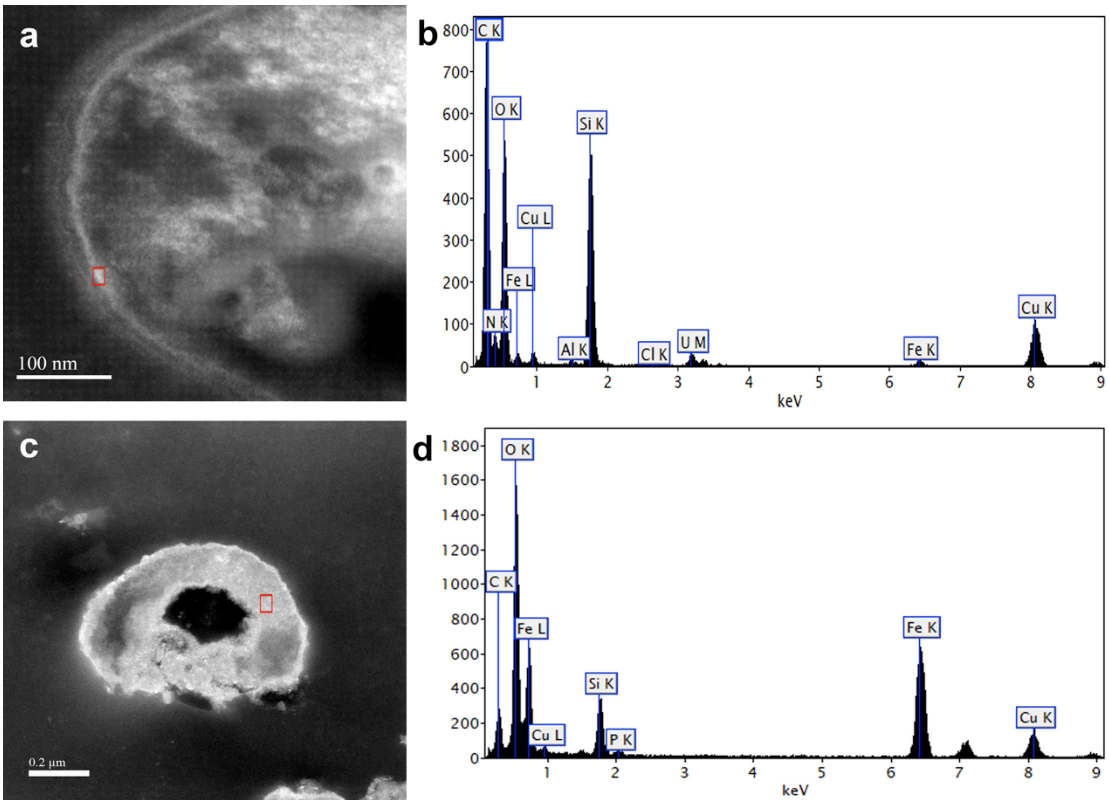

Supplementary Fig. 8. Analytical nanospectroscopy of *M. sedula* grown on NWA 1172.

a, The high angular annular dark field (HAADF) scanning TEM (STEM) image of a cell fragment of *M. sedula* used for electron energy loss spectra (EELS) measurements presented in Fig. 3. **b**, Energy-dispersive X-ray (EDS) spectra acquired from the point indicated on the HAADF-STEM image in (**a**). **c**, HAADF-STEM image of heavily encrusted mineralized cell remnants used for EELS measurements. **d**, Energy-dispersive X-ray (EDS) spectra acquired from the crust of the cell in (**c**).

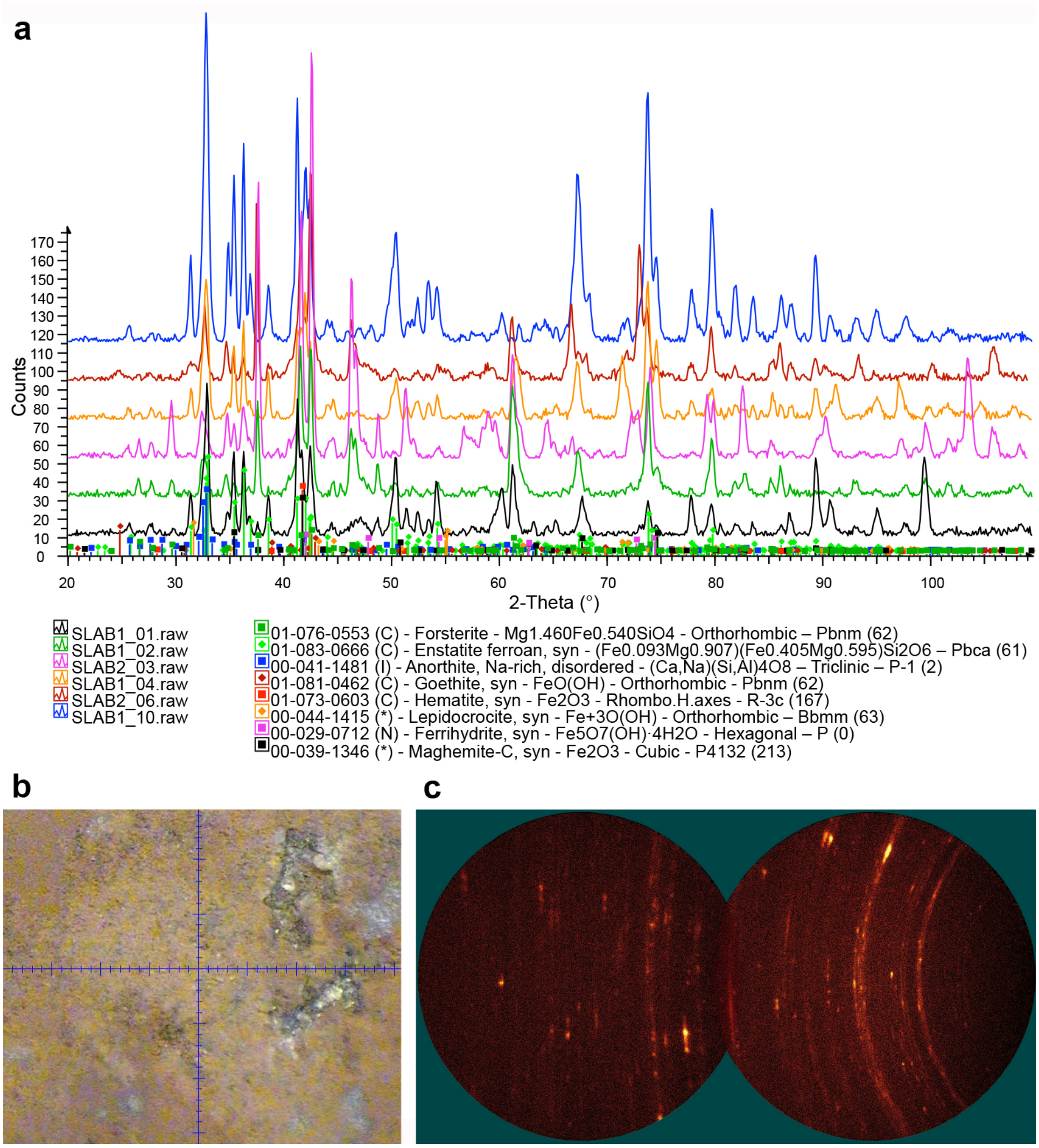

Supplementary Fig. 9. μXRD patterns of the stony meteorite NWA 1172 slab altered by *M. sedula*. a, Integrated diffractogram representing primary and secondary phases detected in NWA 1172 slab fragment after cultivation with *M. sedula*. **b** and **c**, Representative example of photomicrograph and μXRD GADDS images for the *M. sedula* bioprocessed NWA 1172 slab with detected secondary oxide phases: goethite, lepidocrosite, ferrihydrite, and maghemite.

Probe	Sequence 5' - 3' (reverse complementary)	Target gene	Label	Synthesis	Taxon	Target species	FA %	Colour
M.sedula _174_17 mer	AGA UUC CCU UGC CCG CU	16S rRNA	Atto 488	Click chemistry	Archaea	*Metallosphaera sedula*	30%	Green
EUB338	GCT GCC TCC CGT AGG AGT	16S rRNA	Atto 488	Click chemistry	Bacteria	Most bacteria	30%	Green

Supplementary Table 1. Oligonucleotide probes used in this study.

Probe name, nucleotide sequence - underscore indicates nucleotide:fluorochrome conjugates, target gene, label type, label synthesis, target taxon or higher, target species and probe color during imaging.

Composition Results

**Element**	**Shell**	**Comp. (at.%)**
C	K	74.4
N	K	3.7
O	K	10.4
Al	K	0.35
Si	K	5.9
P	K	0.43
S	K	0.13
Cl	K	0.46
Fe	K	0.1
Co	K	0.056
Cu	K	3.6
U	L	0.54

Supplementary Table 2. Percentage elemental composition of *M. sedula* cell grown on NWA 1172 (calculated based on STEM-EDS analysis).

Composition Results

**Element**	**Shell**	**Comp. (at.%)**
C	K	32.9
N	K	0.55
O	K	41.7
Al	K	0.59
Si	K	6.7
P	K	0.66
S	K	0.18
Cl	K	0.34
U	M	0.19
Fe	K	13.5
Ni	K	0.25
Cu	K	2.4

Supplementary Table 3. Percentage elemental composition of *M. sedula* empty envelope encrusted during growth on NWA 1172 (calculated based on STEM-EDS analysis).

Supplementary Video 1:

Motility of *M. sedula* cells grown on the NWA 1172 meteorite as the sole energy source at 73°C after visualization by a modified DAPI fluorescence staining procedure. Recorded with Nikon microscope Jenoptik camera Nikon eclipse 50i microscope with ProgRes® MF cool camera.

Supplementary Video 2:

Motility of *M. sedula* cells grown on chalcopyrite as the sole energy source at 73°C after visualization by a modified DAPI fluorescence staining procedure. Recorded with Nikon microscope Jenoptik camera Nikon eclipse 50i microscope with ProgRes® MF cool camera.
